# International consensus guidance for general population screening for islet autoantibodies to diagnose early-stage type 1 diabetes: a nominal group technique process

**DOI:** 10.1007/s00125-026-06841-z

**Published:** 2026-09-10

**Authors:** Anette-Gabriele Ziegler, Marian J. Rewers, Peter Achenbach, Kirstine J. Bell, Ezio Bonifacio, Linda A. DiMeglio, Sanjoy Dutta, Helena Elding Larsson, Brigitte I. Frohnert, Kurt J. Griffin, William A. Hagopian, Laura M. Jacobsen, Karin Lange, Roberto Mallone, Parth Narendran, Tal Oron, Stephen S. Rich, Caitrin W. McDonough, Darja Šmigoc Schweiger, Zdenek Šumník, Agnieszka Szypowska, Riitta Veijola, Jurgen Vercauteren, John M. Wentworth, Diane K. Wherrett, Anastasia Albanese-O’Neill

**Affiliations:** 1 Institute of Diabetes Research, Helmholtz Munich, German Research Center for Environmental Health, Munich-Neuherberg, Germany; 2 School of Medicine and Health, Forschergruppe Diabetes, TUM University Hospital, Technical University of Munich, Munich, Germany; 3 German Center for Diabetes Research (DZD e.V.), Neuherberg, Germany; 4 Barbara Davis Center for Diabetes, University of Colorado Anschutz Medical Campus, Aurora, CO USA; 5 Charles Perkins Centre and Faculty of Medicine and Health, University of Sydney, Sydney, NSW Australia; 6 Center for Regenerative Therapies Dresden, Faculty of Medicine, Technical University of Dresden, Dresden, Germany; 7 Paul Langerhans Institute Dresden, Helmholtz Centre Munich at the University Clinic Carl Gustav Carus of TU Dresden and Faculty of Medicine, Dresden, Germany; 8 Department of Pediatrics, Indiana University School of Medicine, Indianapolis, IN USA; 9 Breakthrough T1D, New York, NY USA; 10 Department of Clinical Sciences Malmö, Lund University, Lund, Sweden; 11 Department of Pediatrics, Skåne University Hospital, Malmö, Sweden; 12 Benaroya Research Institute, Seattle, WA USA; 13 Department of Pediatrics, Sanford School of Medicine, University of South Dakota, Sioux Falls, SD USA; 14 Dept of Pediatrics, Indiana University, Indianapolis, IN USA; 15 Department of Pediatrics, Division of Endocrinology, University of Florida College of Medicine, Gainesville, FL USA; 16 Medical Psychology, Hannover Medical School, Hannover, Germany; 17 Institut Cochin, CNRS, Inserm, Université Paris Cité, Paris, France; 18 Department of Diabetology and Clinical Immunology, Assistance Publique Hôpitaux de Paris, Cochin Hospital, Paris, France; 19 Indiana Biosciences Research Institute, Indianapolis, IN USA; 20 Queen Elizabeth Hospital, University Hospitals Birmingham NHS Foundation Trust, Birmingham, UK; 21 Department of Immunology and Immunotherapy, University of Birmingham, Birmingham, UK; 22 The Jesse and Sara Lea Shafer Institute of Endocrinology and Diabetes, Schneider Children’s Medical Center of Israel, Petach-Tikva, Israel; 23 Gray Faculty of Medical & Health Sciences, Tel Aviv University, Tel Aviv, Israel; 24 Department of Genome Sciences, University of Virginia, Charlottesville, VA USA; 25 Department of Pharmacotherapy and Translation Research, College of Pharmacy, University of Florida, Gainesville, FL USA; 26 Department of Endocrinology, Diabetes and Metabolic Diseases, University Children’s Hospital, University Medical Centre Ljubljana, Ljubljana, Slovenia; 27 Faculty of Medicine, University of Ljubljana, Ljubljana, Slovenia; 28 Department of Pediatrics, 2nd Faculty of Medicine, Charles University in Prague and Motol University Hospital, Prague, Czechia; 29 Department of Pediatric Diabetology and Pediatrics, Medical University of Warsaw, Warsaw, Poland; 30 Department of Pediatrics, Research Unit of Clinical Medicine, Medical Research Center, University of Oulu, Oulu, Finland; 31 Department for Children and Adolescents, Medical Research Center, Oulu University Hospital, Oulu, Finland; 32 Department of Chronic Diseases and Metabolism, KU Leuven, Leuven, Belgium; 33 St Vincent’s Institute of Medical Research, Fitzroy, VIC Australia; 34 Department of Diabetes and Endocrinology, Royal Melbourne Hospital, Parkville, VIC Australia; 35 Department of Pediatrics, The Hospital for Sick Children, University of Toronto, Toronto, ON Canada; 36 Breakthrough T1D, Gainesville, FL USA

**Keywords:** Consensus report, Early detection, Early-stage type 1 diabetes, Islet autoantibodies, Type 1 diabetes general population screening

## Abstract

Type 1 diabetes is an autoimmune disease that targets and destroys insulin-producing beta cells in the pancreatic islets. The incidence of type 1 diabetes is rising globally. At the clinical diagnosis of type 1 diabetes, between 20% and 67% of children and adolescents present with diabetic ketoacidosis (DKA) requiring hospitalisation, and one-third of these require intensive care. Type 1 diabetes can be detected in early stages, prior to the insulin-requiring clinical diagnosis, through screening for islet autoantibodies (IAbs). Identifying individuals with early-stage type 1 diabetes, combined with monitoring of and education on disease progression, prevents DKA and results in a milder clinical onset. This allows for timely insulin initiation in outpatient settings and improved long-term glucose management. Early diagnosis also enables access to novel disease-modifying therapies that can delay the clinical onset of diabetes. In this international consensus, we provide guidance on the principles and practice of implementing general population screening for IAbs to diagnose early-stage type 1 diabetes. We also outline the minimum requirements for establishing effective population screening programmes to diagnose early-stage type 1 diabetes through IAb detection. This consensus statement has been endorsed by the following professional associations: Advanced Technologies & Treatments for Diabetes (ATTD); Association of Diabetes Care and Education Specialists (ADCES); Association Belge Du Diabète; Associazione Medici Diabetologi (AMD); Australian Diabetes Society (ADS); Belgian Diabetes Liga; Breakthrough T1D; Czech Diabetes Society (ČDS); EASD; Finnish Diabetes Association (FDS); Fondazione Italiana Diabete (FID); International Diabetes Federation (IDF)-Europe; International Society of Paediatric and Adolescent Diabetes (ISPAD); Paediatric Endocrinology Nursing Society (PENS); Polish Diabetes Society; Portuguese Diabetes Association (APDP); Sociedade Portuguesa de Diabetologia (SPD); Società Italiana di Diabetologia (SID); Société Francophone du Diabète (SFD) and Type 1 Diabetes Exchange (T1D Exchange).

**Graphical Abstract:**

**Figure:**
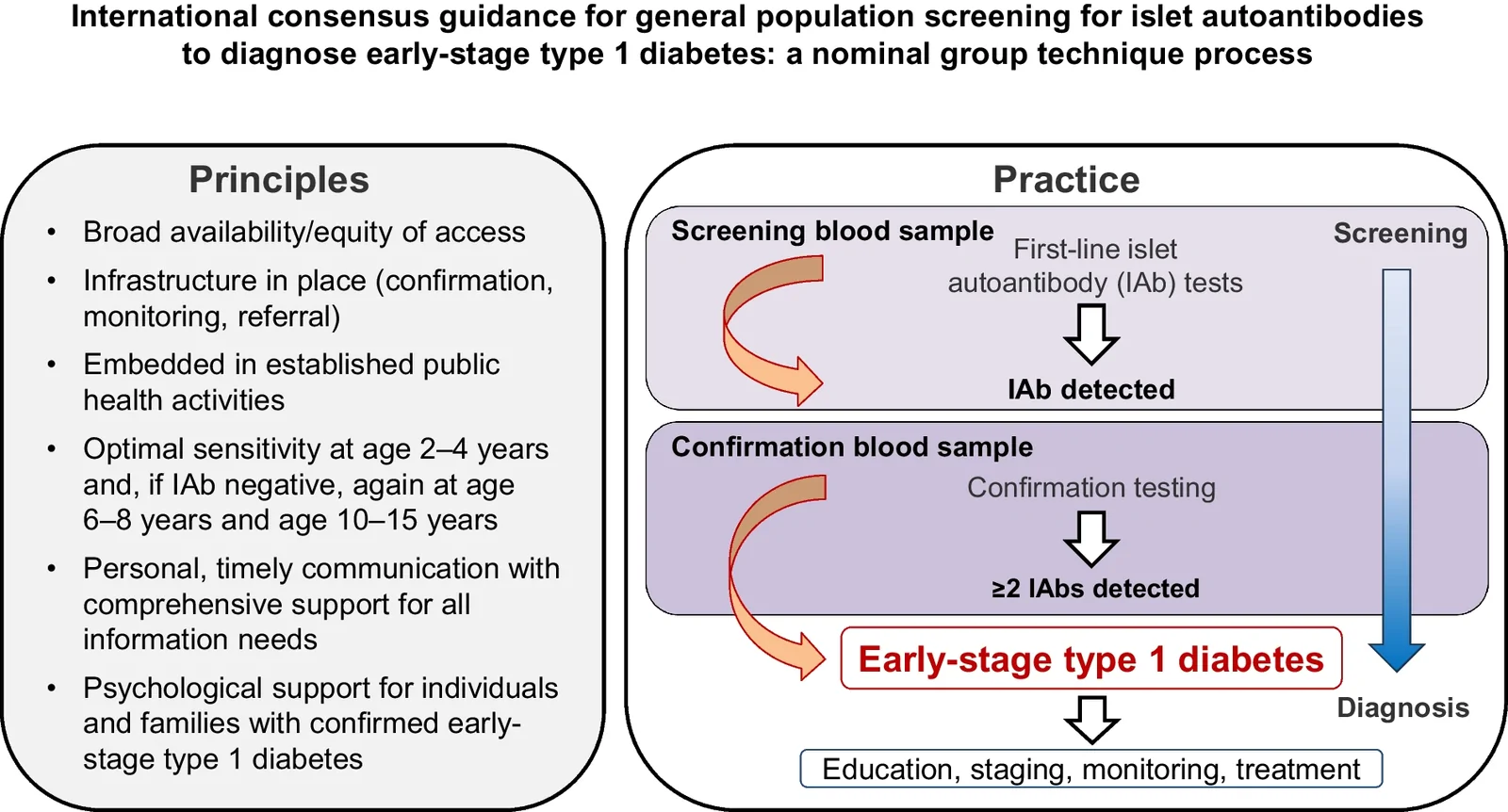

**Supplementary Information:**

The online version contains peer-reviewed but unedited supplementary material available at https://doi.org/10.1007/s00125-026-06841-z.

## Introduction and rationale

Type 1 diabetes is an autoimmune disease in which the immune system targets and destroys insulin-producing beta cells in the pancreatic islets. Symptomatic type 1 diabetes is an important health problem, with annual incidence rates ranging worldwide from 0.2 to 64.2 cases per 100,000 children and adolescents [1]. The incidence is rising by approximately 3% annually [2]. Although type 1 diabetes has been classically defined by the presence of hyperglycaemia that is associated with increased risks for microvascular and macrovascular disease [3, 4], there are recognised early stages of type 1 diabetes during which the underlying autoimmune pathogenesis is detectable but symptoms are absent [5]. Each stage has discernible characteristics (Table 1) [5, 6]: stage 1 is characterised by the presence of two or more confirmed islet autoantibodies (IAbs) with normoglycaemia; stage 2 is characterised by autoimmunity (usually multiple IAbs) and dysglycaemia; and stage 3 is characterised by autoimmunity, hyperglycaemia and usually by overt symptoms, commonly including polydipsia, polyuria, fatigue, weight loss, polyphagia, blurred vision and, particularly in young children, thrush. At present, four IAbs are used clinically to detect early-stage type 1 diabetes (Table 2). It should be noted that islet cell antibodies (ICAs), measured by immunofluorescence assays, have historically been used to identify individuals with IAbs in research settings [7, 8]. However, for the purposes of general population screening, the use of assays directly measuring IAbs to the target antigens listed in Table 2 (insulin, GAD65, insulinoma antigen-2 [IA-2] and ZnT8) are recommended for diagnostic purposes, and ICAs should only be used for research purposes.

**Table 1:** Nomenclature for stages of type 1 diabetes associated with a positive autoantibody screen [5, 6, 52]

Terminology	Stage of type 1 diabetes	ICD-10 code^a^	IAb status	Glycaemic status
At increased risk for type 1 diabetes	Stage 0^b^	E10.A0	Single IAb	Normoglycaemia
Early-stage or presymptomatic type 1 diabetes	Stage 1	E10.A1 or R76	≥2 IAb	Normoglycaemia
	Stage 2	E10.A2 or R76 accompanied by R73.0	Usually ≥2 IAb	Dysglycaemia by ADA criteria [6]
Stage 3 type 1 diabetes (clinical diagnosis)	Stage 3	E10.9	IAb present	Persistent hyperglycaemia by ADA criteria [6] with or without symptoms

^a^E10 Codes defined by Centers for Disease Control in the USA and R73 (abnormal GTT) and R76 codes (abnormal immunological findings in serum) applied by WHO ICD-10 classifications for use outside the USA. Other ICD-10 codes of interest are as follows: family history of type 1 diabetes (Z83.3); personal history of diseases of the blood and blood-forming organs and certain disorders involving the immune mechanism [autoimmune disease] (Z86.2); and screening for diabetes mellitus (Z13.1)

^b^As defined by TrialNet

**Table 2:** Autoantibodies used to detect islet autoimmunity and early-stage type 1 diabetes

Autoantibody	Antigen specificity	Characteristics
IAA	Insulin	Common as a first detected IAb in young children [141, 142] More common in younger children [109]
GADA	GAD	Common in children, adolescents and adults with early-stage type 1 diabetes [141–143] Not specific to islet cell autoimmunity or early-stage type 1 diabetes [144, 145]
IA-2A	Tyrosine phosphatase islet antigen-2	Associated with a faster progression to stage 3 type 1 diabetes [122, 123]
ZnT8A	ZnT8	Presence can improve risk stratification in individuals with single GADA, IAA or IA-2A [146]

Often, a type 1 diabetes diagnosis is first made following presentation to the emergency department or admission to hospital with diabetic ketoacidosis (DKA), which may be life-threatening in children at onset of type 1 diabetes [9] and can have serious acute and chronic consequences, including the development of acute kidney injury (AKI) due to circulatory volume depletion [10, 11]. DKA at diagnosis is also associated with longer-term issues for metabolic control, including elevated HbA_1c_ compared with individuals with type 1 diabetes but no or mild DKA at diagnosis [12–14] as well as increased risk of subsequent episodes of DKA [15, 16]. Importantly, even single episodes of DKA have detectable neurocognitive consequences [17, 18]. The incidence of DKA at diagnosis of type 1 diabetes in children and adolescents varies considerably by region [19–24] with recent data indicating rates of 20% in Sweden [19], 21–36% in Germany [24], 41% in Australia [25] and 35–58% in the USA [26, 27]. Younger age of children and adolescents has been associated with higher risk of DKA at diagnosis in some studies (OR 1.59–1.80) [21, 28], whereas data from a Finnish study has reported higher rates in children 10–14 years old (26.4%) at pubertal age, compared with children aged ≤4 years (16.5%) or 5–9 years (14.6%) [29]. DKA event rates of 23% at diagnosis have been reported for adults aged 18–45 years [30]. Population-based studies [19, 21, 28] have correlated younger age, not having a relative with type 1 diabetes, race/ethnicity minority status and poor health insurance status as predictors of DKA at diagnosis. Age-related DKA risk is clearly more complex than age alone; however, youth with these associated demographic characteristics may particularly benefit from screening, early detection and monitoring.

Early detection and diagnosis of early-stage type 1 diabetes by IAb screening and participation in monitoring programmes markedly reduces the incidence of DKA at stage 3 type 1 diabetes, often to below 5%. For example, at one US diabetes centre, monitored children with early-stage type 1 diabetes had a DKA rate of 4.9%, compared with 48.5% in the regional general population [31]. Even those not enrolled in active follow-up showed partial benefit, with an intermediate rate of 21.4% [31]. Similarly, the Fr1da general population screening study in Germany reported a DKA rate of 3.2% among children with early-stage type 1 diabetes who were monitored [32, 33], compared with national rates of 21–36% [24]. In Italy, regional pilot programmes for islet IAb screening significantly reduced DKA incidence in young children compared with the incidence in unscreened regions, providing evidence that increased general awareness about type 1 diabetes, especially by physicians, played a key role [34]. The Environmental Determinants of Diabetes in the Young (TEDDY) study has followed at-risk participants aged from <2 years to 15 years. Among those aged 3 months to 5 years, incidence of DKA at stage 3 type 1 diabetes diagnosis was reduced to 15% compared with national rates of 29–54% reported in countries where TEDDY participation was unavailable [35].

Decades of research has investigated therapies to induce immune tolerance, protect beta cells and prolong endogenous insulin production. Teplizumab was approved by the US Food and Drug Administration (FDA) in November 2022 [36], by the UK Medicines and Healthcare products Regulatory agency (MHRA) in August 2025 [37], with marketing authorisation issued by the European Commission on 8 January 2026 [38]. Health Canada gave teplizumab marketing approval in May 2025 but Canada’s Drug Agency has recommended it not be reimbursed [39]. Teplizumab has been shown to delay progression of stage 2 type 1 diabetes to stage 3 type 1 diabetes by a median of 2.7 years [40], thus extending the time until insulin therapy is required [41]. Limited real-world outcomes of teplizumab treatment also show that progression of stage 2 type 1 diabetes is stabilised or reduced over 6 months of follow-up [42], and the impact of using teplizumab in real-world clinical practice is increasingly the focus of discussion [43]. Approval of this therapy marks a paradigm shift in type 1 diabetes clinical care, introducing immunological therapies in addition to metabolic therapies to the clinical toolkit. Clinical trials are underway to identify other agents that can delay type 1 diabetes disease progression [44].

The goals of general population screening for the presence of IAbs and diagnosis of early-stage type 1 diabetes encompass reducing the prevalence of DKA at presentation of stage 3 type 1 diabetes, improving care for individuals at all stages of type 1 diabetes, reducing healthcare costs associated with DKA and management of type 1 diabetes, offering disease-modifying therapy as early as possible, and providing access to clinical trials aimed at delaying disease progression.

In this international consensus document our aim is to provide evidence-based guidance for general population screening for IAbs to detect and diagnose early-stage type 1 diabetes, with the expectation that this should be applicable to ongoing systematic screening programmes, to programmes in development, or to on-demand testing for individuals who may not have participated in screening at a recommended time. In this context, we provide clear and actionable recommendations on current best practices, each of which have been graded according to the ADA system for evidence appraisal (electronic supplementary material [ESM] Table 1) and are aligned with the WHO’s ‘Short Guide to Screening Programmes’ [45].

## Methods

Breakthrough T1D, a non-governmental not-for-profit organisation headquartered in the USA, invited healthcare professionals (HCPs) with expertise in detection of early-stage type 1 diabetes, using screening for IAbs prior to onset of clinical symptoms, to participate in a consensus panel. The consensus process was designed to follow a nominal group technique (NGT) methodology, combining brainstorming, discussion and refinement of ideas, to reach consensus [46]. The NGT methodology is designed to allow each participant to voice their views at each distinct stage of the process, and to agree or disagree within the overall group structure. The consensus process was not prospectively registered. The consensus panel comprised opinion leaders drawn from academic institutions and various diabetes, paediatric and primary care associations globally. All authors of this review are members of the consensus writing group. People living with diabetes and family members outside of academia were also invited to contribute. Participation in the writing group was voluntary and was not remunerated. An initial one-day workshop was hosted in Paris (on 20 November 2024), at which topics of relevance to the overall objective of general population screening for IAbs were discussed. After this activity, six working groups were incorporated to provide informed feedback on the issues of most relevance, with appropriate evidence to support all discussion points. Each working group held virtual meetings moderated by an independent participant. Each session was structured to begin with, with each member of the group talking through their own specific insights and ideas regarding the consensus. Following these contributions, a group discussion was conducted to identify common points for consideration as consensus items. All virtual meetings were recorded in order that a full review could be undertaken at any point. Once each working group had contributed in this manner, a draft summary of outcomes was circulated containing the consensus recommendations for review. There were 12 rounds of manuscript review and development. At the end of this process, a blinded online voting step was undertaken from 10 September 2025 to 12 September 2025, stating each consensus recommendation with the option to agree or disagree. An 80% approval rate or more was set as the adoption threshold. All consensus panelists responded and all recommendations within this consensus met the ≥80% threshold.

### Screening for early-stage type 1 diabetes: definition

When screening for type 1 diabetes is considered, often genetic screening and IAb screening are both discussed. In clinical trials and observational studies, IAb screening for detection of early-stage type 1 diabetes has focused on children and adolescents with a defined genetic risk, either as first-degree relatives of an individual with symptomatic type 1 diabetes, or carrying defined high-risk HLA DR/DQ haplotypes, or with a high type 1 diabetes genetic risk score. As such, learned societies such as the ADA included IAb screening of these individuals into their Standards of Care [6]. However, more than 85% of individuals with newly diagnosed stage 3 type 1 diabetes lack a family history [47, 48], which underlines the utility of extending IAb-based screening programmes to the general population [32, 49, 50].

In this consensus guidance, genetic screening to detect risk of type 1 diabetes is not addressed. Genetic screening identifies people at risk of developing autoimmune type 1 diabetes [51], while IAb screening detects the presence of type 1 diabetes as a disease, albeit at an early stage [52]. Independently of the screening strategy, each of the early-stage type 1 diabetes classifications were assigned specific ICD-10 diagnosis codes in 2024 applicable in the USA (Table 1) and the WHO ICD-10 classifications R76 (abnormal immunological findings in serum) and R73 (abnormal GTT) applicable outside of the USA (Table 1). These should support access and reimbursement for interventions appropriate to each stage. At this time, general population IAb screening may be more effective and practical than genetic risk-based strategies for identifying early-stage type 1 diabetes and reducing complications such as DKA, especially when considering real-world implementation challenges such as recall rates, the complexity of informed consent and potential parental concerns around genetic screening [32, 51, 53]. This consensus guidance is focused on detecting early-stage type 1 diabetes in the general population through screening for IAbs, including available cost-effectiveness determinations for such screening based on immediate screening costs and long-term health economic benefits, as discussed later.

It is widely accepted that testing for IAbs should be used for accurate classification of diabetes type at the time of diagnosis [54, 55]; however, such differential diagnosis for people with diagnosed diabetes is not part of this consensus statement and will not be addressed. For additional guidance, refer to published clinical guidelines related to the diagnosis and classification of diabetes [6].

### Observational and longitudinal studies that provide the current evidence

Evidence from large early-stage type 1 diabetes screening programmes has informed these consensus recommendations. These include the Type 1 Diabetes TrialNet Pathway to Prevention study [56, 57], the Type 1 Diabetes Prediction and Prevention (DIPP) Study [58], TEDDY Study [59], the BABYDIAB study [60], DAISY [61, 62], the DiPiS study [63], the DiaUnion/TRIAD study [64], the Type 1 Diabetes Intelligence (T1DI) Consortium [65], the Fr1da study [32, 66] and ASK study [67]. Both the Fr1da and ASK early type 1 diabetes studies implemented general population screening and provide valuable estimates about the frequency of early-stage type 1 diabetes and the population benefits and costs, including DKA reduction, milder disease onset, lower long-term HbA_1c_, more residual insulin production and fewer hospital admissions (see Benefits and potential harms of early detection).

## Benefits and potential harms of early detection

Application of the WHO principles that underpin the value of public health screening is compelling in the case of early-stage type 1 diabetes (see Text box, ‘Wilson and Jungner criteria for public health screening for disease’) [68]. Early-stage type 1 diabetes detection has significant benefits (Table 3). These include the opportunity for education for symptom awareness and monitoring of disease progression, which can markedly reduce the incidence of DKA at onset of hyperglycaemia [32, 33, 69]. Early diagnosis and management of glucose levels also improve treatment outcomes following the start of insulin therapy [70, 71]. The wider benefits of general population IAb screening are also described in Table 3. These are evidence-based outcomes that collectively have the potential to significantly improve the immediate and short-term health and wellbeing of the individual who is diagnosed with early-stage type 1 diabetes, with associated benefits for parents and caregivers, and reduced costs for healthcare services through reduction in DKA and the need for hospitalisation at clinical diagnosis [32, 67, 72]. For individuals with detected IAbs, most of these benefits are closely linked to participation in ongoing monitoring programmes [31, 58].

**Figure:**
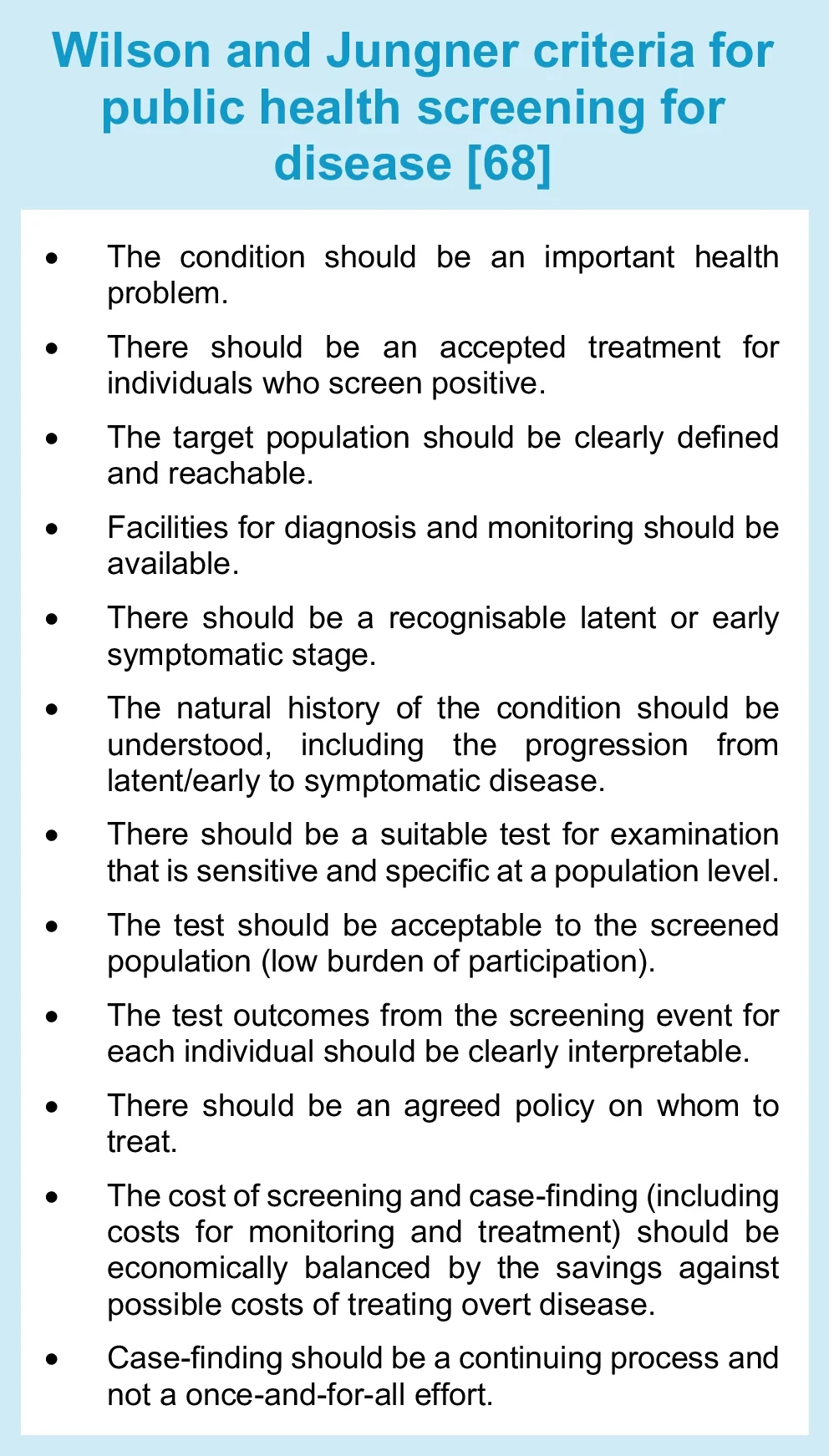

**Table 3:** Benefits and potential harms of general population screening for IAbs

Benefits of screening prior to clinical diagnosis	Benefits of screening at clinical diagnosis and later	Potential harms of screening
• Metabolic monitoring allows estimation of time to onset of stage 3 T1D [147]. • Access to tailored diabetes management education for IAb-positive individuals and their families gives time to plan and prepare for a ‘soft landing’ [95, 148]. • Opportunity to start disease-modifying therapy in IAb-positive individuals for whom access to these therapies is possible, to preserve beta cell function and delay the need for insulin therapy [41]. • Opportunity to participate in clinical trials of novel interventions to stop or slow progression to insulin dependence [30, 40, 95].	• Significantly reduces the occurrence of DKA in IAb-positive individuals and its acute neurological, vascular and kidney complications [11, 13, 17]. Although rates vary, rate reductions in DKA of >85% have been reported [31, 58]. • Reduced hospitalisation rates at onset of symptoms with consequent reduction in associated direct and indirect costs, which can mitigate screening programme costs [67, 72, 140, 149]. • Early diagnosis leads to milder disease at clinical presentation with lower HbA_1c_ and higher C-peptide levels [33]. • Early detection and management of hyperglycaemia in T1D is associated with improved long-term metabolic control [70]. • Improved quality of life for affected individuals and their families at the time of clinical diagnosis and following initiation of insulin therapy [95].	• Blood sampling can be painful for the individual, with risks for bruising. Can be mitigated by use of topical anaesthetics. • Psychosocial impact: awareness of early-stage T1D may induce immediate and long-term anxiety, although this can decrease over time or with participation in a monitoring programme [137, 150]. • False-positive test result: this can cause anxiety and depression, even if corrected. Reinforces the need for highly specific IAb assays to minimise false-positive results. • False reassurance following a negative screen, indicating the need for clear guidance and information on communicating results. • Negative behaviour changes, such as very low carbohydrate dieting in young children, excessive exercise or vitamin supplementation, none of which have been shown to delay the progression of T1D. • A diagnosis of early-stage T1D can have consequences in situations where social stigmatisation is known to occur [74]. Increased general awareness and education regarding early-stage T1D can counter this.

T1D, type 1 diabetes

The potential harms of screening are summarised in Table 3. Several relate to misperception of risk for progression to stage 3 type 1 diabetes, as well as anxiety, depression or restrictive dietary or behavioural changes. It is possible to identify these consequences and ensure that support is provided. Accurately predicting progression to clinical stage 3 type 1 diabetes remains challenging for individuals with confirmed early-stage disease following IAb screening and glycaemic assessment, although multivariable models offer population-specific 5 year rate projections that can be used to visualise individual risk estimates [73]. A confirmed diagnosis of early-stage type 1 diabetes may also lead to stigmatisation or discrimination in healthcare, insurance, education, employment and social settings [74], all of which require proactive intervention [75, 76]. While the costs of implementing general population screening are a consideration, available cost–benefit analyses suggest favourable long-term health economic outcomes [67, 72]. Successful programmes must therefore minimise potential harms while maximising the benefits of early diagnosis and delayed progression to stage 3 type 1 diabetes.

## Mitigation of potential harms

Public awareness of type 1 diabetes remains limited. Therefore, education and communication in conjunction with the screening effort are essential. Results from the Early Surveillance for Autoimmune Diabetes (ELSA) project showed that positive parental attitudes were linked to clear explanations of screening goals, differentiation between type 1 diabetes and type 2 diabetes, and prior experience with type 1 diabetes or other public health programmes [77]. A systematic review of blood-spot screening in newborn babies also emphasised the importance of timely, transparent communication in parental acceptance [53]. In the Fr1da study, most parents of children diagnosed with early-stage type 1 diabetes were positive about their decision, with 84% satisfied or very satisfied that their child participated in screening for IAbs, and 80% assessed their decision to participate as good or very good [78]. Similarly, a qualitative study [79] based on interviews with parents from families with a history of symptomatic type 1 diabetes, who had chosen to have their child screened for IAbs, expressed generally positive experiences, whereas non-participants had a negative view of screening, often based on self-directed research. Further insights come from the UNISCREEN pilot study in Italy, which evaluated screening for type 1 diabetes and coeliac disease [80]. Among 1535 participants, most expressed satisfaction with the process and 90% preferred simple capillary fingerstick sampling over venous collection.

## Populations: who to screen

IAb screening to diagnose early-stage type 1 diabetes should be offered to the general population. Most individuals who develop stage 3 type 1 diabetes do not have a prior family history of the disease and are at particularly high risk of presenting with DKA [81]. Evidence demonstrates that general population screening and detection of early-stage type 1 diabetes improves metabolic outcomes, reduces DKA incidence, smooths the transition to insulin therapy, and altogether leads to a milder disease onset and less anxiety at clinical onset [32, 33]. Furthermore, children with early-stage type 1 diabetes who do not have a prior family history of type 1 diabetes progress to stage 3 type 1 diabetes at rates comparable with those of children with a family history or prior genetic risk [66, 82–84]. IAb screening in the general population represents a critical step towards equitable care, enabling access to follow-up, monitoring and emerging disease-modifying therapies to all with early-stage type 1 diabetes.

Depending on the local context, resources and public acceptance, some regions may choose to begin general population screening with individuals potentially at increased risk of early-stage type 1 diabetes, such as relatives of people with symptomatic type 1 diabetes or other autoimmune diseases, individuals with personal history of other autoimmune diseases, or those identified as genetically at risk through genetic screening programmes. Evidence shows that having a first-degree relative with symptomatic type 1 diabetes increases the lifetime risk of developing stage 3 type 1 diabetes by up to 15-fold, compared with the unselected general population [32, 85]. Additionally, a parent with autoimmune thyroid disease, coeliac disease, rheumatoid arthritis, pernicious anaemia, sarcoidosis, mixed connective tissue disease or Sjögren’s syndrome has been associated with increased risk of stage 3 type 1 diabetes in the offspring [86]. A known genetic association with selected HLA DR/DQ haplotypes confers a 5- to 10-fold increased risk for early-stage type 1 diabetes [87], and individuals with combined HLA and non-HLA genetic risk scores may have up to 25-fold increased risk for early-stage type 1 diabetes [88]. There is also a bidirectional association between other pre-existing autoimmune diseases and risk for developing stage 3 type 1 diabetes, including coeliac disease [89, 90], autoimmune thyroiditis [89, 91], vitiligo and systemic lupus erythematosus (SLE) [92], Addison’s disease, polymyalgia rheumatica, multiple sclerosis, myasthenia gravis and pernicious anaemia [93].

### Policy and infrastructure

When establishing a regional or national general population screening programme, the oversight may come from a regional or central authority or healthcare administrator. An example of such a programme is in Italy, where in 2023 the Italian Parliament approved a law that mandated screening for early-stage type 1 diabetes and coeliac disease should be offered to all children and adolescents, and be managed through the National Health System (SSN). The implementation phase involved a ‘propaedeutic’ pilot study, conducted by the Istituto Superiore di Sanità (ISS), with initial outcomes indicating significant reductions in DKA rates at onset of stage 3 type 1 diabetes as a consequence of increased general public and physician awareness about clinical type 1 diabetes [34]. Other examples include regional implementation of screening, sponsored and approved by relevant associations of healthcare providers with the objective of achieving the framework contracts with healthcare payers [49, 94]. Critically, the moment the screening for early-stage type 1 diabetes is adopted by a healthcare system, it should specify the requirement for a developed infrastructure for follow-up confirmation and monitoring for those with detectable IAbs, for which clinical guidance has been published [95].

### Experience from general population screening of children and adolescents – ongoing initiatives

To date, a national public health screening for early-stage type 1 diabetes in general paediatric populations has been governmentally mandated only in Italy [96], where screening is still in a preliminary pilot phase to assess sustainability [97]. The Fr1da study in Bavaria, Germany, was initiated as a regional public health research screening programme for children aged 1.75 to <6.0 years, screening once between 2015 and 2019 [32, 49]. It was subsequently extended to screening children aged 1.75 to <11 years, with two screenings, 3 years apart, in order to increase detection rates [33, 66, 82]. Families of children who have confirmed two or more IAbs are invited to participate in a programme of diabetes education, metabolic staging, assessment of psychological stress and prospective monitoring for progression of their type 1 diabetes status. Of the >200,000 individuals screened for IAb between 1 February 2015 and 31 July 2024, 0.30% (*n*=576) had early-stage type 1 diabetes [82]. Of those who had metabolic staging (*n*=435), 71% (*n*=309) had or developed stage 2 type 1 diabetes during follow-up; the 2 year risk of developing stage 3 type 1 diabetes was 32.2% for those meeting the ADA stage 2 definition [82]. It is worth noting that children in the Fr1da study identified by general population screening have a similar rate of disease progression when compared with those with a family history of symptomatic type 1 diabetes and a genetic risk [32, 66, 83]. An important component of this study was that 66.4% of the primary care paediatricians in Bavaria participated in the initiative and the screening test was conducted during a scheduled visit for a well-baby check, an established public health activity [32]. Since its initiation, the Fr1da study has been extended to other regions in Germany, including Saxony, Lower Saxony, Hesse and Rhineland-Palatinate, showing that general population screening and monitoring is scalable, sustainable and relatively low cost in the context of its scope [72].

The ASK study [44] screened for IAbs in 34,110 children aged 1–17 years from 2017 to 2023. Participant age, sex, and race/ethnicity matched a Colorado population demographic. Only 6% of participants had a first-degree relative diagnosed with type 1 diabetes. Most participants were screened in paediatric clinics and outpatient labs. Type 1 diabetes autoantibodies, including IAA, GADA, IA-2 autoantibody (IA-2A) and ZnT8A, as well as transglutaminase antibodies to detect coeliac disease or coeliac autoimmunity, were measured using both standard radiobinding assay (RBA) and a more specific electrochemiluminescence (ECL) assay, distinguishing predictive IAbs (high-affinity) from non-predictive ones (low-affinity) [98, 99]. Screened children with detectable IAbs were invited to a confirmation visit that included measurement of IAbs by RBA and ECL, HbA_1c_, random blood glucose and detailed review of symptoms. At the initial screening or confirmation, 0.54% of the children had multiple IAbs (stage 1 type 1 diabetes) and a further 0.42% had a single high-affinity IAb (RBA^+^ and ECL^+^). During a 3 year follow-up, on average, progression to stage 3 type 1 diabetes was observed in 29% of children with multiple IAbs and in 6.3% of those with a single high-affinity IAb [100]. Among children who were confirmed with early-stage type 1 diabetes, 4.5% had DKA at diagnosis, compared with 58% reported for unscreened Colorado children [27].

Following the Fr1da model, the EDENT1FI consortium has published a Master Protocol [101] that covers public health screening for the presence of IAbs in children and adolescents, including staging using OGTTs, elevated HbA_1c_, random glucose or continuous glucose monitoring for IAb-positive participants. Significantly, the protocol also makes recommendations for education, monitoring and follow-up of children screened positive for early-stage type 1 diabetes and their families. General population screening for IAb is ongoing or planned in the Czech Republic, Germany, Denmark, Italy, Poland, Portugal, Sweden and the UK, with at least 200,000 individuals aged 1–17 years expected to be screened over the 5 year study period, either using independent methodologies or following the EDENT1FI Master Protocol [101].

### Preliminary data from general population screening of adults

More than half of cases of new-onset stage 3 type 1 diabetes are diagnosed in adults [102]. Data from the ASK study indicate that adults (mean age 41 years) screen positive for multiple (two or more) IAbs at a rate similar to or higher than the rate in children and adolescents (0.6% adults vs 0.4% youth) [103]. In this study, 1.7% of adults also screened positive for a single high-affinity IAb, compared with 0.5% of youth. At the time of enrolment, 10% of paediatric and adult participants reported a family member with type 1 diabetes but this was not an exclusion criterion. It should be noted that in other studies the prevalence of IAbs in adults with normal glucose tolerance was low, with no predictive value for progression to future stage 3 type 1 diabetes [104]. However, this study was based on data obtained in 1990 when the overall prevalence of islet autoimmunity was lower than it is currently. Moreover, it considered only GADA and IA-2A, so is limited by the absence of data on other IAbs, such as IAA and ZnT8A, used for detecting early-stage type 1 diabetes. More recent data from the T1D TrialNet study have also suggested adults with a family history of type 1 diabetes are more likely than children to screen for a single IAb (4.0% vs 2.6%) but have a lower prevalence of multiple IAbs (0.83% vs 2.8%) [105]. However, adults with stage 2 type 1 diabetes at initial staging (using OGTT) had comparable 5 year risk of progression to stage 3 type 1 diabetes as children (78% for each group) [105].

The prevalence of DKA at diagnosis of stage 3 type 1 diabetes in adults is significant [30, 106]. The Type 1 Diabetes Risk in Adults (T1DRA) study [107] in the UK has undertaken general population screening in adults up to 70 years of age, with self-collection and return of fingerstick capillary blood samples via postal kits. T1DRA is aligned with the ELSA childhood screening study [108]. Each initiative aims to screen at least 20,000 individuals.

### Consensus recommendations

- Where policy and infrastructure is in place, IAb screening for early-stage type 1 diabetes should be offered to the general population. [C]
- Targeted screening of individuals who are at increased risk due to personal or family history of autoimmune disease may be a practical starting point for screening but the ultimate goal is general population screening. [C]
- Screening awareness communications should emphasise the benefits of timely detection of early-stage type 1 diabetes. [B]
- Prior to starting general population screening for early-stage type 1 diabetes, there must be a developed infrastructure for (1) confirmation of positive results and (2) monitoring for those with early-stage type 1 diabetes, including referral pathways between primary and secondary care, and between paediatric and adult services, as needed. [C]

## Age and cadence for general population screening for islet autoantibodies

Among children genetically at risk followed from birth to stage 3 type 1 diabetes, the incidence of development of IAbs was highest between the ages of 9 months and 3 years, and decreased rapidly after age 6 years [60, 109, 110]. A two-times screening strategy, first at age 2–3 years, then a second at age 5–7 years, fits with established primary care well-baby and school-readiness visits or vaccination schedules. The T1DI study group used harmonised data from the DIPP, BABYDIAB, DiPiS, DAISY and DEW-IT studies to assess a two-age screening strategy for 6722 children from first inclusion in each registry to 15 years of age or until diagnosis of stage 3 type 1 diabetes [65]. The study found that the optimal age for single-age IAb screening was 4 years but that sensitivity and positive predictive value (PPV; see Text box, ‘Using IAb screening results to inform progression to symptomatic type 1 diabetes’) was optimal when a two-age screening strategy was used, once at approximately 2 years of age and again at approximately 6 years of age. These observations concur with the data from the TEDDY study, which included children with the highest HLA-conferred genetic risk [110], and with the Fr1da study, which included children from the general population [51, 69, 111]. A second analysis by the T1DI study group concluded that a one-time IAb screening at age 10 years was highly predictive of developing stage 3 type 1 diabetes by age 18, with a sensitivity of 90% and a PPV of 66% [112].

**Figure:**
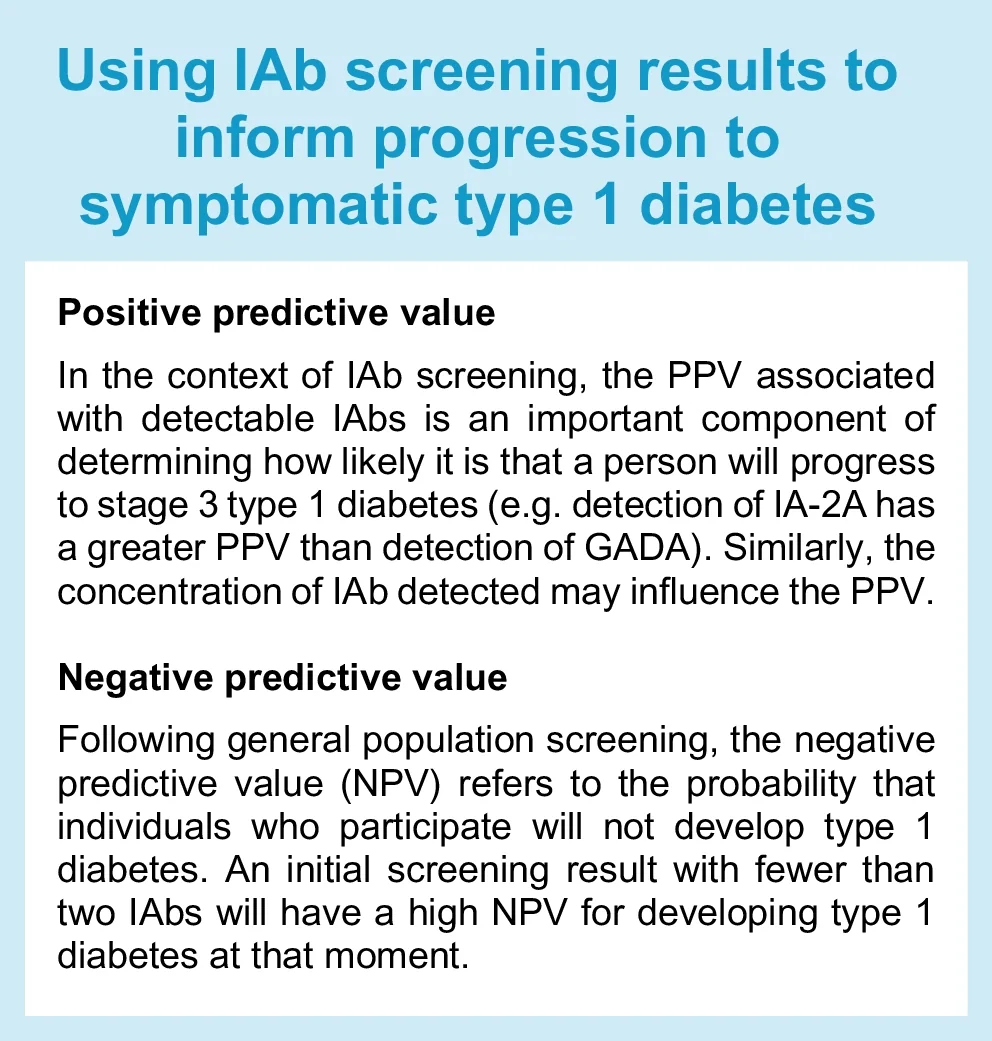

In the general population of adults, aged 18 years and older, there are limited data to inform an optimal age or frequency of IAb screening. The TrialNet study and others [113, 114] have reported that adults with a family history of type 1 diabetes, who have been identified as IAb positive, exhibit slower progression to stage 3 type 1 diabetes requiring insulin [113], with a 5 year rate of 15% compared with 35% for children aged 12 years or younger. Overall, there is an unmet need for more data on the frequency and progression of IAb screening in general population adults.

### Consensus recommendations

- General population screening at age 2–4 years and, if IAb-negative, again at age 6–8 years and at age 10–15 years is recommended for detecting individuals with early-stage type 1 diabetes. Children who have never been screened should catch up by joining this schedule at the earliest opportunity [B]. For any screening, the age ranges indicated can be adapted to the screening opportunities provided within established public health activities. [E]
- Less is known about early-stage type 1 diabetes progression in the general population older than 15 years. If not done earlier, a one-time screening after 15 years of age may be considered in conjunction with established public health activities. [E]

## General population screening: the steps

Overall, an effective IAb screening programme is a stepwise process, which funnels a large number of individuals through a series of decision gates that each increase confidence in the final outcome. The process starts with an initial screening for IAb and concludes with metabolic staging assessments and the diagnosis of early-stage type 1 diabetes. These steps are outlined in the Text box, ‘The flow of testing in general population screening for early-stage type 1 diabetes’.

**Figure:**
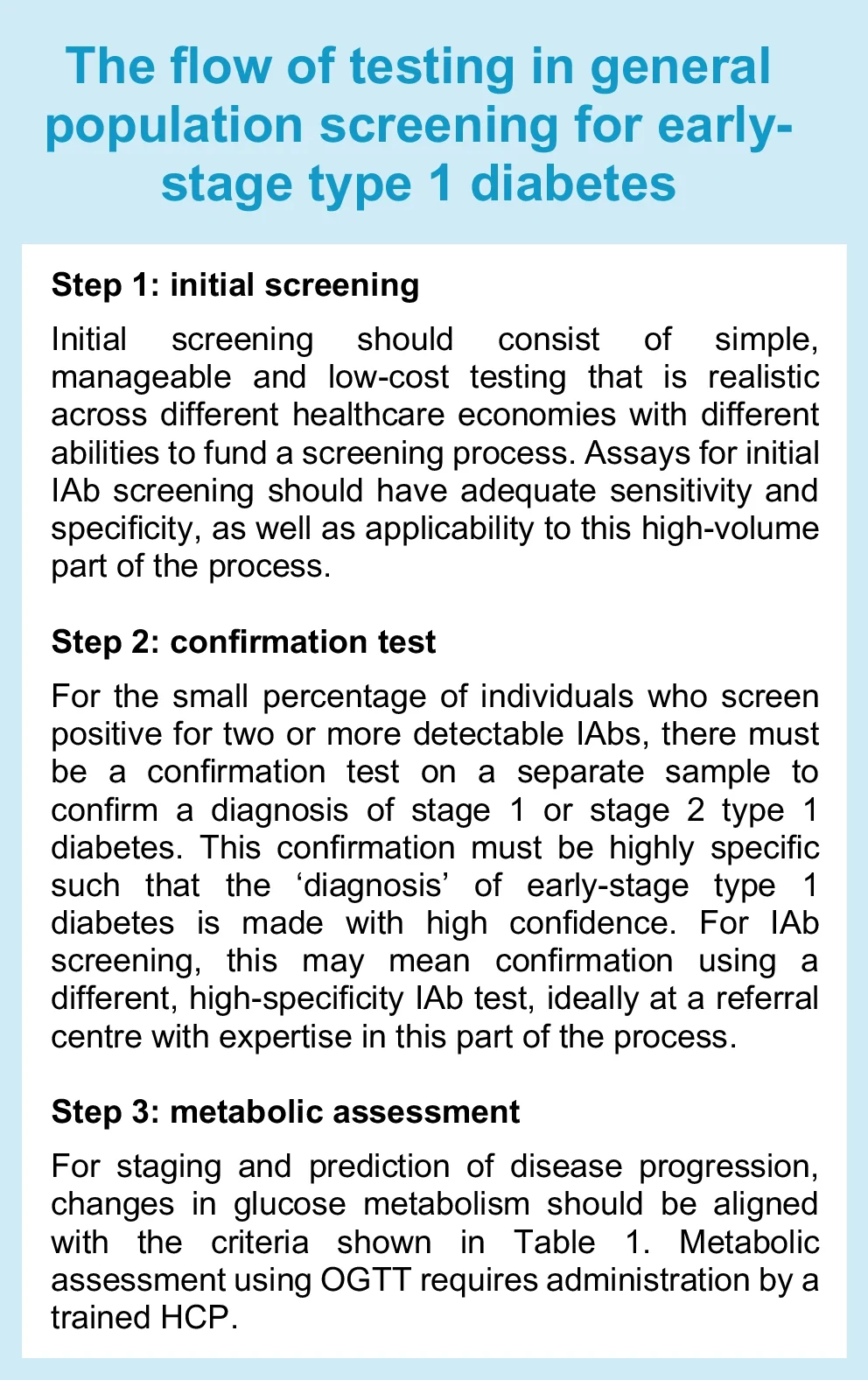

### Participation, context and setting

Experience with diverse public health initiatives indicates that optimising participation requires a low burden for the person to be screened, including for parents of screened children. The Fr1da general population screening study made sample collection part of a business-as-usual visit to a family paediatrician for a routine well-baby check-up [32, 49]. This had the benefit of also minimising the burden for HCPs. Equally important is adequate reimbursement for the work involved in screening, as well as for maintaining a reference centre that coordinates screening-related activities, including materials, information, shipments, results reporting and recommendations for appropriate follow-up. With the availability of multiplex reagents and small-volume assays, the opportunity also exists to screen for several autoimmune diseases at once, with little extra burden for the participant, including screening for type 1 diabetes, autoimmune thyroiditis or coeliac disease [64, 98, 99, 115, 116].

The opportunities to incorporate IAb screening as part of a ‘business-as-usual’ public health activity may include well-baby and well-child clinics, and early childhood vaccination programmes (e.g. the measles, mumps and rubella triple vaccine), each of which are part of a visit to a healthcare clinic or through a home-visit by an HCP. These existing public health activities are convenient for family members and leverage established expectations, although there must be capacity available for additional communication and screening tasks. It is also important to demonstrate that they do not compromise participation in the existing public health activities. Ideally, IAb screening should not be an ad-hoc event, adding to the burden of participation, and must ensure equity of access across all individuals, unrestricted by socioeconomic status, race/ethnicity, geography or health insurance status [44, 45, 117]. The screening must also be part of a structured activity that is supported for this purpose. Integration in this way can optimise the participation of primary healthcare teams. For school-age children, vaccination programmes managed in the school setting may provide an opportunity to combine with IAb screening. A qualitative, proof-of-concept as part of the T1Early study [118] found that parents of children screened for IAbs during attendance at an established preschool vaccination visit, were universally positive about the combined public health scheduling. Significantly, the group indicated that they would have been less likely to participate in screening if it was separate from the visit to the vaccination clinic.

For adults, the options to combine participation can include routine blood checkups and health checks for elevated glucose, cholesterol or hypertension [119]. Each of these checks can be delivered in primary care settings. Workplace employee health screening is now also routine for many individuals, with the goal of improving workforce health and productivity. Both for adults and for children, the use of postal kits for self-sampling and return of samples is also seen as viable [64, 107].

The ideal setting (or settings) for IAb general population screening will depend on local practices and capacity for public health initiatives, and their ability to adapt and include IAb screening. In the European EDENT1FI project, which focuses on children in the general population [101], blood sample collections for screening are performed by primary care physicians and paediatricians or paediatric hospitals, during preventive medical checkups, other paediatric visits, hospital stays and through school or home tests. Participation in screening programmes is not expected to be mandatory. For any person offered and encouraged to participate, it is important that there is a clear and informed mechanism for opting in or opting out of the screening process; this mechanism may differ depending on the location and operational boundaries of the prevailing healthcare system. Whatever the setting, the requirement for active follow-up and monitoring must be met for all individuals with confirmed IAb positivity, and must be built in to the general population screening infrastructure.

### Consensus recommendations

- IAb testing should be embedded in established public health activities that minimise the barriers to participation and leverage existing structures. These can include, but are not limited to, vaccination clinics and well-child clinic appointments. It is expected that different healthcare services will adopt the screening context that is most appropriate to their regional public health capacity and process. [E]
- IAb screening should promote equity of access for all individuals, independent of socioeconomic status, ethnicity or regional location. [B]
- Whenever and wherever screening is offered, the provider (the person managing the screening visit) should be knowledgeable on key points relevant to the screening process, including the components of pre-screening awareness and information, the screening event, the delivery of results and any necessary referral process. It is expected that adequate reimbursement for these activities is available. [E]

## Assays

**Fig. 1:**
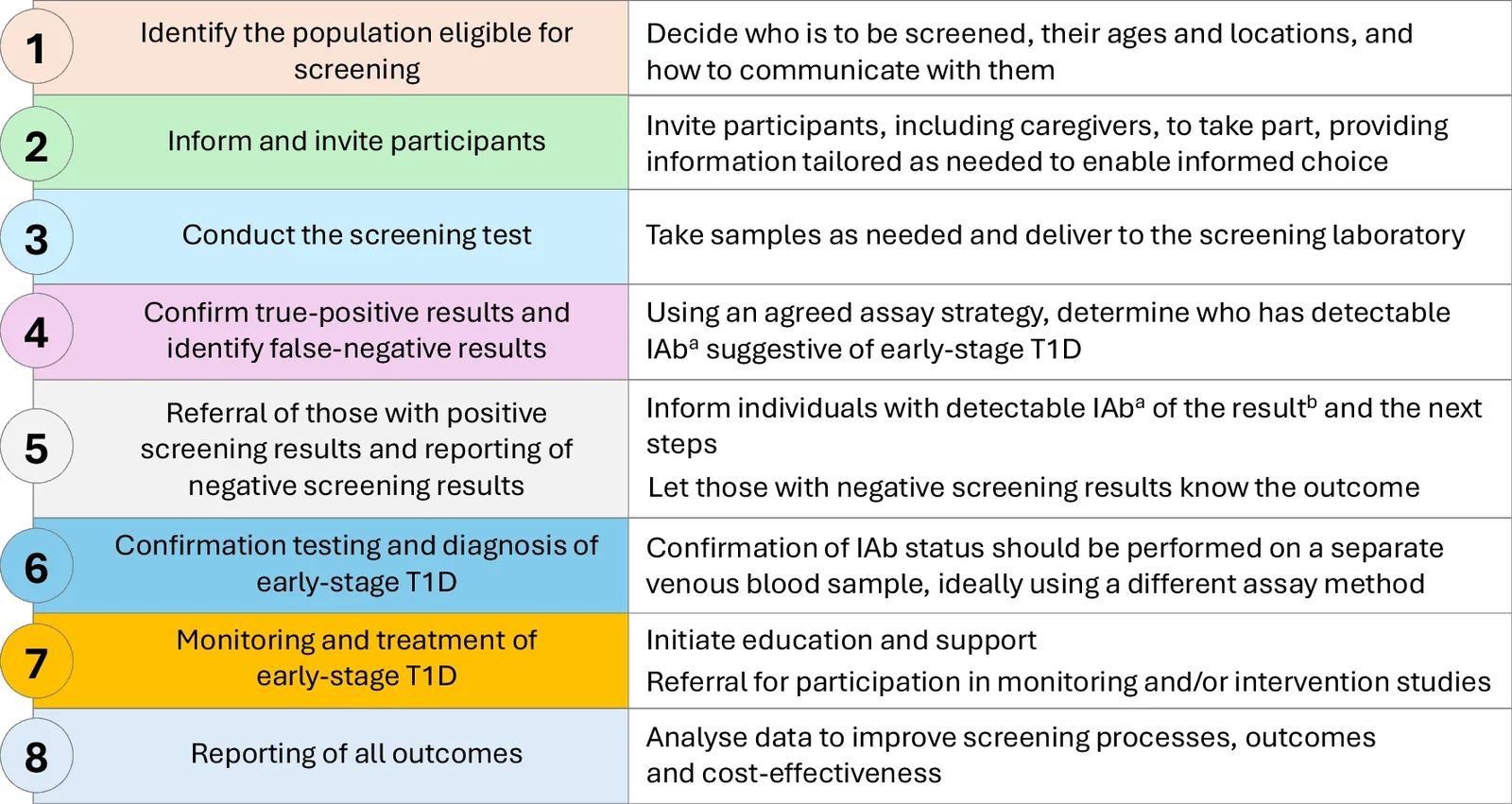
The flow of actions in a general population IAb screening programme. ^a^Two or more IAbs detected. ^b^Personal contact required, not automated emails, texts or pro-forma letters. T1D, type 1 diabetes

Public health screening programmes are a process leading to the diagnosis of early-stage type 1 diabetes [45, 111]. The steps in this process are described briefly in Fig. 1. For steps 3–5, it is possible that a central reference laboratory, with capacity for high-throughput screening, can manage this part of the process, including, shipping tests and collection. To date, a series of assay modalities have been used in IAb detection, including RBA, ELISA, ECL assay, luciferase immunoprecipitation (LIPS), antibody detection by agglutination-PCR (ADAP) and chemiluminescence assay (CLIA). These are described in Table 4. For general population screening, assay modalities should be approved and available outside of research settings, and accessible by providers with the required understanding of the implications of a positive or negative screen. Assays should also be validated in the Islet Autoantibody Standardization Program (IASP) [120]. Since the diagnosis of early-stage type 1 diabetes requires the presence of at least two of the four IAbs, screening assays should measure at least three of the four major IAbs. Although data are limited across diverse racial and ethnic study populations, if only two IAbs are used for the initial screening test, the use of IAA and GADA is likely to provide the highest sensitivity for detecting early-stage type 1 diabetes in children of European ancestry.

**Table 4:** Available IAb detection assays

Assay modality	Detection methodology	Advantages	Disadvantages
RBA	Radiolabelled autoantigens form complexes with IAb All IgG is captured and bound antigen is quantified with scintillation counters [151]	Gold standard for IAb measurement with established standard protocols and performance [120] High sensitivity Low serum volume required for GADA, IA-2A, ZnT8A Reagents generally freely available without commercial licensing for the majority of IAbs Semi-automation can achieve high throughput in experienced labs	Higher sample volume required for IAA Requires radioisotopes and associated regulation and therefore is only likely to be available in large institutions with existing licences Increased cost from required frequent radiolabelled autoantigen production and from the lack of full automation Not easily multiplexed
Luciferase immunoprecipitation (LIPS)	Luciferase-labelled autoantigens are exposed to IAb sample All IgG is captured and bound antigen is quantified by luciferase enzymatic reaction producing bioluminescence measured in a luminometer [152]	High sensitivity Low serum volume Amenable to high-throughput automation Multiplex potential (without discrimination of which IAbs are positive)	Not commercially available Relies on in-house reagents that are less widely available and likely need individual lab standardisation Multiplex assay does not distinguish which antibody is positive
ECL bridge assay	Autoantigens labelled with a plate capture tag and a detection electroluminescence tag are exposed to IAb sample in liquid phase Autoantigen–IAb complexes are captured on a solid-phase electrode When electrical potential is applied the tagged complexes emit light [153]	High sensitivity Commercially available Amenable to high-throughput automation Multiplex potential to screen and identify all four IAbs	Multiplex assay requires higher volume than some other assays Reliance on proprietary commercial reagents and instrumentation increases cost
Bridge ELISA	IAb sample is exposed to plate bound autoantigens Biotin-labelled autoantigens are added and captured on the solid phase, followed by quantification by addition of streptavidin peroxidase and a colorimetric signal [154]	High sensitivity Although proprietary, it is commercially available at relatively low cost Used in several research screening programmes Amenable to high-throughput automation Multiplexed (without discrimination of which IAbs are positive)	Does not include IAA Higher volume than some other assays Multiplex assay does not distinguish which antibody is positive
Dissociation-enhanced lanthanide fluorescent immunoassay (DELFIA), a bridge enzyme-immunosorbent assay	IAb sample is exposed to plate bound autoantigens Biotin-labelled autoantigens are added and captured on the solid phase^a^ Quantification is via the addition of streptavidin peroxidase followed by anti-peroxidase antibodies labelled with multiple europium molecules and enhanced time-resolved fluorescence readout [155]	Designed for DBS requiring very low blood volume Based on colorimetric bridge ELISA Commercially available Amenable to high-throughput automation Multiplexed (without discrimination of which IAbs are positive)	Limited data on performance Limited application in research and real-world studies Does not include IAA Reliance on proprietary commercial reagents and instrumentation with uncertain costs Multiplex assay does not distinguish which antibody is positive Additional steps to bridge ELISA
Antibody detection by agglutination-PCR (ADAP)	DNA-labelled autoantigens are exposed to IAb sample to form complexes A bridging oligonucleotide then hybridises with the DNA from the antigens, and DNA ligase joins the strands to create a PCR-amplifiable DNA sequence This is then quantified using quantitative PCR [156]	High sensitivity Low serum volume required DBS assay available Commercially available Amenable to high throughput Multiplex potential to screen and identify all four IAbs	Reliance on proprietary commercial reagents and instrumentation increases costs
Chemiluminescence assay (CLIA)	Sample is added to autoantigens bound to nano-magnetic beads IAbs bind and are detected by chemiluminescence-labelled protein A or anti-human IgG and substrate-induced light emission [157]	Amenable to high-throughput automation Multiplex potential (without discrimination of which IAbs are positive)	Limited data on performance Potentially larger sample volume due to fluidics dead volume Reliance on proprietary commercial reagents and instrumentation with uncertain costs

^a^Although the assay employs a biotin-conjugated antigen, its performance is unlikely to be affected by excess serum biotin, as biotinylated antigen is added only after serum removal

Initial screening tests should be relatively inexpensive, highly sensitive and able to process a large number of samples. Testing of samples from the general population, where disease prevalence is low, requires that thresholds for positivity be aligned to the need for higher sensitivity (i.e. find as many individuals with an IAb), with the impact of potentially lowered specificity (i.e. an increased rate of false positives) [121]. Screening tests are, therefore, first-line tests that trigger further analyses, which must include confirmatory tests. Cost of screening and confirmation is also a relevant consideration. Since confirmatory tests are usually more expensive than screening tests, a high frequency of samples and individuals requiring confirmatory assessments should be avoided. Confirmatory testing requires assays that can distinguish positivity for all four IAbs. This can be achieved with single IAb assays or some of the multiplexed assays (Table 4). For a number of the assays, there is limited information as to their standardisation and performance in large-scale cross-sectional population screening. Furthermore, not all IAbs are equivalent: the detection of IA-2A (Table 2) has a higher PPV (see Text box, ‘Using IAb screening results to inform progression to symptomatic type 1 diabetes’) for progression to stage 3 type 1 diabetes compared with other IAbs [66, 122–124]. IAA is less frequent in adults and its detection may not be required if the assay is not readily available [103].

### Minimising false-positive and -negative diagnoses: the rule of 2s

One way to mitigate the potential for a high rate of false positives is to specify that diagnosis of early-stage type 1 diabetes requires positivity for at least two separate IAbs, using two assay methods, in two consecutive samples (2 × 2 × 2). Screening studies of high-risk children indicate that reversion from multiple to single IAb status is relatively rare [125]. However, this does not necessarily apply in cross-sectional population screening, hence the recommendation for additional layers or confirmation.

The value of using two different assays is that each assay should detect positive signals associated with the presence of IAbs in different ways (Table 4), each with different vulnerabilities for false positives. Thus, testing each sample across two assay methods (a screen and validation strategy) enables the positive signals from the first assay to be validated in the second assay, with reduced occurrence of false positives that are associated with each assay. Confirmation in a second sample is important to reduce false diagnoses due to sample quality or handling, as well as transient elevations in assay signals. Notably, the EDENT1FI Master Protocol for public health screening has adopted a 2-Cube assay strategy similar to the approach used in the Fr1da study [101, 115].

If constraints limit the application of a 2 × 2 × 2 approach, the minimum requirement is a 2 × 2 modality that detects two IAbs at two different time points. Separately, the IASP has defined assay performance standards that can be used to assess performance of separate IAb detection assay modalities performed on serum or plasma [120].

Requirements for the rule of 2s approach as part of general population screening are detailed as follows:

- Screening assays that are sensitive and detect at least three of four IAbs (i.e. they identify most individuals with early-stage type 1 diabetes)
- Screening assays that are sufficiently specific (e.g. >97%) to allow reasonable and cost-effective second line testing
- A validation assay(s) that is different in methodology (Table 4), and with sensitivity for detection of similar rates of true IAb signals compared with the first screening assay
- A validation assay(s) that detects and distinguishes each of the four IAbs
- Confirmation of IAbs should be performed on a separate venous blood sample
- Assays should require small volumes and have minimal ‘dead volume’ that cannot be used by the testing equipment
- Clinical testing laboratories should have a proven track record in IAb screening (e.g. participation in proficiency testing programmes such as IASP)
- All assays should be available with adequate distribution, long-term supply and reasonable costs.

Limitations of the rule of 2s approach also include the following:

- As with most strategies meant to increase specificity, a strict application of the 2 × 2 × 2 approach is likely to generate more false-negative results
- In some healthcare systems, availability of IAb assays may be limited
- The requirement to be positive for two or more IAbs at two time points will generate a number of ‘grey zone’ results when either only one visit was completed or the individual was positive for only one IAb at confirmation or for different IAbs at screening vs confirmation. Therefore, screening programmes should have a protocol for appropriate risk assessment, results communication, and follow-up of participants who fall in this ‘grey zone’.

### Sample type

The type of test sample is an important consideration. Serum and plasma are optimal for current testing modalities, either from venous or capillary compartments. Where possible, these samples are recommended. It is also possible to detect IAbs both in venous and capillary whole blood or in dried blood spots (DBSs), which have been used for screening tests [126–129]. Fingerstick capillary blood sampling using at-home sample kits, with return of DBS test cards, has also been shown to be acceptable to participants in IAb screening [128]. However, data on whole blood and DBS are fewer than for serum and plasma, and standardisation and quality control (QC) programmes currently only include serum or plasma measurements of IAbs. Furthermore, the volume of blood required for DBS measurements is often similar to that required for serum or plasma measurement. While home and whole blood or DBS testing may be feasible for screening, confirmatory testing should be performed on serum or plasma obtained from venous blood.

### Consensus recommendations

- Test sample collection and delivery to a clinical laboratory should maximise participation and promote equitable access to screening among underserved groups based on socioeconomic status, geography or ethnicity. [C]
- The IAb detection assay protocols for general population screening for early-stage type 1 diabetes must mitigate against high numbers of false-positive and false-negative tests. [B]
- At the point of confirmation, the diagnosis of early-stage type 1 diabetes must have a high degree of confidence in the IAb detection assay protocols and procedures employed. [B]
- The selected IAb assays should be certified and validated for use outside of the research study context. [E]
- When IAbs are detected during screening, confirmation with a second blood sample is required. [B]
- Confirmatory testing should ideally use a different assay method from that used in the initial screening test, when available. [E]

## Communication associated with effective general population screening

Research studies confirm that IAb screening and identification of early-stage type 1 diabetes in children, with subsequent participation in education and monitoring, is associated with improved outcomes compared with age-matched children diagnosed with incident type 1 diabetes without prior screening [32, 33]. This supports the immediate and long-term value of general population screening and communication of these messages is a critical part of pre-screening awareness, to encourage engagement with follow-up [79, 130].

### General principles

All participants or their primary caregivers should know both the purpose of IAb screening and what they can expect to happen during the screening process, including practical information about what happens immediately following the screening event. All terminology should be accessible to the general population and use language that encourages engagement, including the meaning of having early-stage type 1 diabetes and the high value of preventing DKA. Information on potential interventions and/or clinical trials with disease-modifying therapies should be tailored to the availability of the intervention/trial at the time of screening. The separate needs for communication before and in the immediate post-screening period are detailed in Table 5.

**Table 5:** Key information and education needs before and after screening for IAbs

Prior to screening	Following an IAb screen
Key goal is to encourage participation in screening: • What is the purpose of screening? • What does it involve? • Where and when will the screening take place? • What is type 1 diabetes and why is it good to know your status? • What is DKA and why is it important to avoid it? • What will happen if the screening test indicates early-stage type 1 diabetes? ○ Clinical support available immediately, if needed. ○ Psychological and emotional support available. ○ Educational support available. ○ Access to regular monitoring of glucose metabolism. ○ Access to disease-modifying therapies and intervention trials where available.	If fewer than two IAbs are detected: • This indicates a low concern for development of T1D at this time but please note that this does not mean that T1D will never develop. Since IAb status prior to screening is not known, rescreening may be advised. If two or more IAbs are detected, provide education and information on the following: • What the test result means right now. • What does it mean for the future? • Importance of confirmation and staging, and what the stages mean. • Probability for progression of type 1 diabetes. • Monitoring glucose levels, how and how often. • Awareness of the symptoms of hyperglycaemia. • Principles of healthy lifestyle (nutrition, physical activity) while avoiding severe restrictive or excessive behaviours. • When insulin therapy might be needed. • What are the available treatment options, including diabetes technology? • What are the available disease-modifying therapies and clinical trials? • Contact information for the diabetes clinic.

### Following a screening test detecting a single IAb

Individuals with a screening test detecting only a single IAb do not meet the criteria for early-stage type 1 diabetes, and a high proportion of those with a single IAb revert to being antibody negative during follow-up [125]. However, those with a confirmed single IAb can have an increased risk of developing multiple IAbs, or progressing to stage 3 type 1 diabetes, particularly if they are younger or have a known genetic predisposition or a first-degree relative with type 1 diabetes, when compared with individuals who do not have an IAb on screening [114, 131, 132]. Clinical guidance indicates that children aged 3 years or less at the time of single IAb detection should be monitored for at least 3 years [95, 133]. It is also important to recognise that individuals who screen positive for multiple IAbs may lose one or more of the IAbs in the progression to stage 3 type 1 diabetes and thereby may present as only single IAb positive on a cross-sectional screening event. The predictive value of a single IAb test result in the general population without previous IAb monitoring, a positive family history or elevated genetic risk remains unknown. Therefore, individuals with a single IAb should not be considered diagnosed with early-stage type 1 diabetes. They may be followed within the context of research studies [95], which is done in ASK [67], in the Fr1da study [32] and in the Australian Type 1 Diabetes National Screening Pilot [94].

### Following a screening test detecting two or more IAbs

Communicating the outcome of a screening test should promote understanding and minimise confusion. For example, the terms ‘positive’ and ‘negative’ can be misinterpreted, conveying both good and bad outcomes. The primary goal of communicating the outcome is to inform the person and their family (if appropriate) that early-stage type 1 diabetes is suspected and that a confirmatory test is needed. The importance of a confirmatory test should be discussed, along with the importance of participating in monitoring programmes and considering participation in intervention studies, if available [95]. In the event that a confirmatory test is negative (i.e. does not show IAbs), it is equivalent to a negative screening result and this news can be provided as part of the now-established thread of contact, assuming that there is no reason to doubt the conduct of the test. Providing helpful information and support is key to encouraging healthy coping with the diagnosis and ongoing engagement in follow-up monitoring and care. This should be done with a personal contact (including by phone call) by a provider possessing the knowledge to answer any questions or concerns that the individual and carers may have. An important outcome of the Fr1da study organisation and delivery was that 85% of parents were satisfied or very satisfied with the in-person communication of the diagnosis of early-stage type 1 diabetes by their primary care provider [78]. Ideally, a positive screening result, irrespective of multiple or single IAb status, should not be first revealed as part of an automated clinical test service or electronic medical record update. Depending on the screening setting, where feasible, some details regarding the specifics of the initial screening result should not be disclosed until a confirmation test has been discussed, including the types of IAb confirmed and any estimation of progression.

### Following a screening test detecting no IAbs

A screening result that does not detect an IAb indicates that early-stage type 1 diabetes is not suspected. Communicating this outcome must clearly reinforce that the result indicates a low level of concern for development of stage 3 type 1 diabetes at this time but that this does not mean the individual is never going to develop stage 3 type 1 diabetes. This is also an important time to highlight the need for IAb rescreening in the future. This conversation is particularly important in young children and if there is family history of type 1 diabetes or other forms of autoimmunity, where the risk of seroconversion is higher. There are several options for communicating that early-stage type 1 diabetes is not suspected. These include written confirmation to the screened individual or their caregivers, letters to primary care services for them to follow-up, addition of information to a person’s electronic medical record, or follow-up by phone call. It is also important to summarise the common symptoms of hyperglycaemia and the need to immediately contact their HCP if these symptoms appear. This is particularly important for the caregivers of young children who may develop positivity for IAbs and progress to clinical type 1 diabetes quite fast despite no IAbs being detected in the initial screening test.

## Psychosocial support

As with other diagnoses, communication of a diagnosis of early-stage type 1 diabetes can cause an expected grief response, distress and negative emotions [134]. The Fr1da study monitored psychological stress for 6 and 12 months after confirmation of early-stage type 1 diabetes in children and found that 1.5% of parents reported symptoms of anxiety and depression [49], a rate comparable with that among the general population in Germany. The TEDDY study has reported parental anxiety related to glucose monitoring in children with established genetic risk of type 1 diabetes [135, 136], and this was more likely in mothers than in fathers. At the first ASK follow-up population-based monitoring visit, parental anxiety was higher than in the TEDDY study and elevated above the clinical cutoff of 40 (State Anxiety Inventory [SAI] score 46.1). At the following visit, anxiety remained elevated but started to decrease (*p*=0.03) after a median 6.1 months [137]. Aspects of education and psychological support for individuals (as well as their caregivers) who have received confirmation of a positive IAb screen have been well documented in previous consensus guidance for monitoring of early-stage type 1 diabetes (stage 1 and stage 2) [95]. The available insights into communication, education and psychosocial support around the IAb general population screening process are largely derived from the Fr1da study [32, 78], with additional strategies and outcomes from prospective studies of children and adolescents with known risk factors for stage 3 type 1 diabetes.

An attitudes survey among parents of children participating in the ELSA general population screening project [77] indicated that the parents found participation in screening more acceptable when informed they would be provided with psychosocial support should their child screen positive. A survey of families of children with newly diagnosed clinical stage 3 type 1 diabetes [138] indicated that, in the immediate period following a positive screen, families value diabetes education (75%), contact with the diabetes team (72%), and support from family (50%) and friends (24%). Communication with individuals and families must place emphasis on empathetic communication with consistent easy-to-understand information and practical self-management actions (if any), along with age-appropriate information for children. Critically, as mentioned previously, the results of the IAb screening test should not be revealed to the participant prior to their being contacted for confirmation testing. Since many healthcare services are obligated to share all clinical test results with their patient populations without delay, we acknowledge that there is unlikely to be a standard approach to this and individual healthcare systems will have their own strategies for minimising participant anxieties. Whether it is needed or not, the availability of psychosocial support can be factored into general population screening, although, to date, no association has been reported between IAb detection on screening for early-stage type 1 diabetes and a formal diagnosis of depressive disorder.

A key constituent for education in the context of general population IAb screening is primary care HCPs and teams. This group will have considerable responsibility for managing at least part of the process, especially if screening activities are aggregated into established primary care services (e.g. vaccination clinics, well-baby clinics). Secondary care diabetes services also need to be an integrated part of the screening process, and general population screening initiatives should be prepared to ensure that individuals with confirmed early-stage type 1 diabetes can expect to be provided care within the most appropriate section of the healthcare service. A selection of resources that illustrate the overall management of an IAb screening programme is provided in the Text box, ‘Resources available from early-stage type 1 diabetes autoantibody research. In this context, the focus should be on the detection of pathological distress reactions and persistent depression in those screened or their care providers, with prompt psychological support being offered in those cases.

**Figure:**
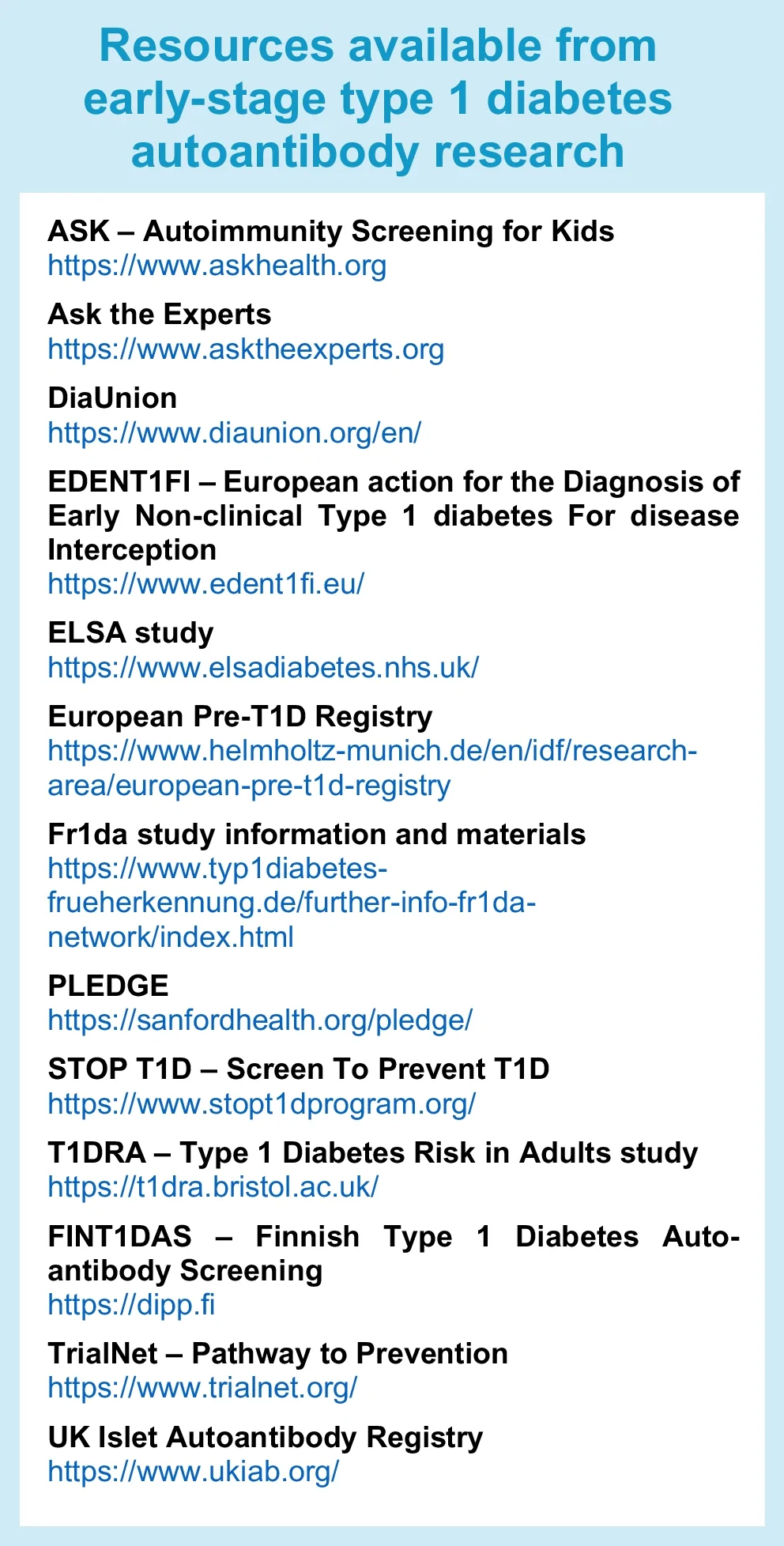

### Consensus recommendations

- Communications planning before and after the immediate IAb screening activity is the responsibility of national or regional stakeholders. At minimum, communication should clarify both the purpose of IAb screening and what can be expected at the screening appointment and in the immediate period thereafter. [C]
- Use clear and neutral terminology when communicating a screening test result. Until a screening result that detects the presence of IAb is confirmed, it is important to avoid language that creates avoidable anxiety for the screened individual. [E]
- When communicating screening and confirmatory test results to the individual or caregiver, a personal contact should be offered to allow for the explanation of results. [E] All individuals should have the opportunity to express their immediate needs for information and support. [B]
- Communication that IAbs were not detected must emphasise that the current result does not preclude type 1 diabetes in the future and rescreening may be recommended, particularly if the person is young or has a personal or family history of autoimmune disease. [C]
- If a single IAb is detected, future rescreening may be recommended based on age and other factors. [B]
- All screening participants should be provided with clear information about identifying the symptoms of type 1 diabetes. [E]
- HCPs should be aware of psychological reactions to the delivery of a diagnosis of early-stage type 1 diabetes, in order to take actionable steps (e.g. provide information/education, referral and/or monitoring plan). When necessary, professional psychological support should be offered to reduce anxiety. [C]

## Cost-effectiveness of screening

Depending on the prevailing healthcare system in a given region, each step of the workflow must be systematised and reimbursable for the process to be scalable and sustainable. Anticipated costs for screening to participants and healthcare services are summarised in the Text box, ‘Anticipated costs of general population screening for early-stage type 1 diabetes’. Only a small number of cost-effectiveness analyses have been undertaken for general population screening for early-stage type 1 diabetes. In one of these, based on 10,029 children and adolescents from the ASK study in Colorado [67], screening was assessed as being cost-effective, at a value threshold of US$50,000–150,000 per quality-adjusted life year (QALY), if it both lowered the rate of DKA at diagnosis of stage 3 type 1 diabetes in the screened population by 20% (e.g. from 35% to 28%) and also improved HbA_1c_ by −1.1 mmol/mol (−0.1%) over a lifetime. Cost savings were increased at higher rate reductions of DKA and greater improvements in HbA_1c_. Against this assessment, real-world DKA reductions of more than 43% have been reported across study cohorts in Colorado [31], although long-term HbA_1c_ data are not yet available. The screening cost per child in the ASK study cohort was US$47–141 and the screening cost per case of early-stage type 1 diabetes detected was US$4700. A costing analysis of the outcomes from the Fr1da study in Bavaria, Germany [32], applied as standard of care for Germany rather than the Fr1da cohort alone, estimated a cost of €21.73 per screened child and €7035 per case detected [72]. More limited simulation studies have suggested that break-even costs for IAb screening programmes can be predicted if unit costs per IAb test were US$25–40 and onset of type 1 diabetes was delayed by 3−5 years [139], although the input data in these models included participants from genetic risk studies, which is a limitation for conclusions regarding general population screening. One study has reported that the costs of genetic and IAb screening would need to be extremely low to make screening cost-effective for prevention of DKA at diagnosis in children younger than 5 years [140]. This was despite cost savings against treatment and hospital admissions, as well as lost parental income. Overall, more longitudinal real-world data are needed to understand the full immediate and long-term costs and benefits of general population screening for early-stage type 1 diabetes.

**Figure:**
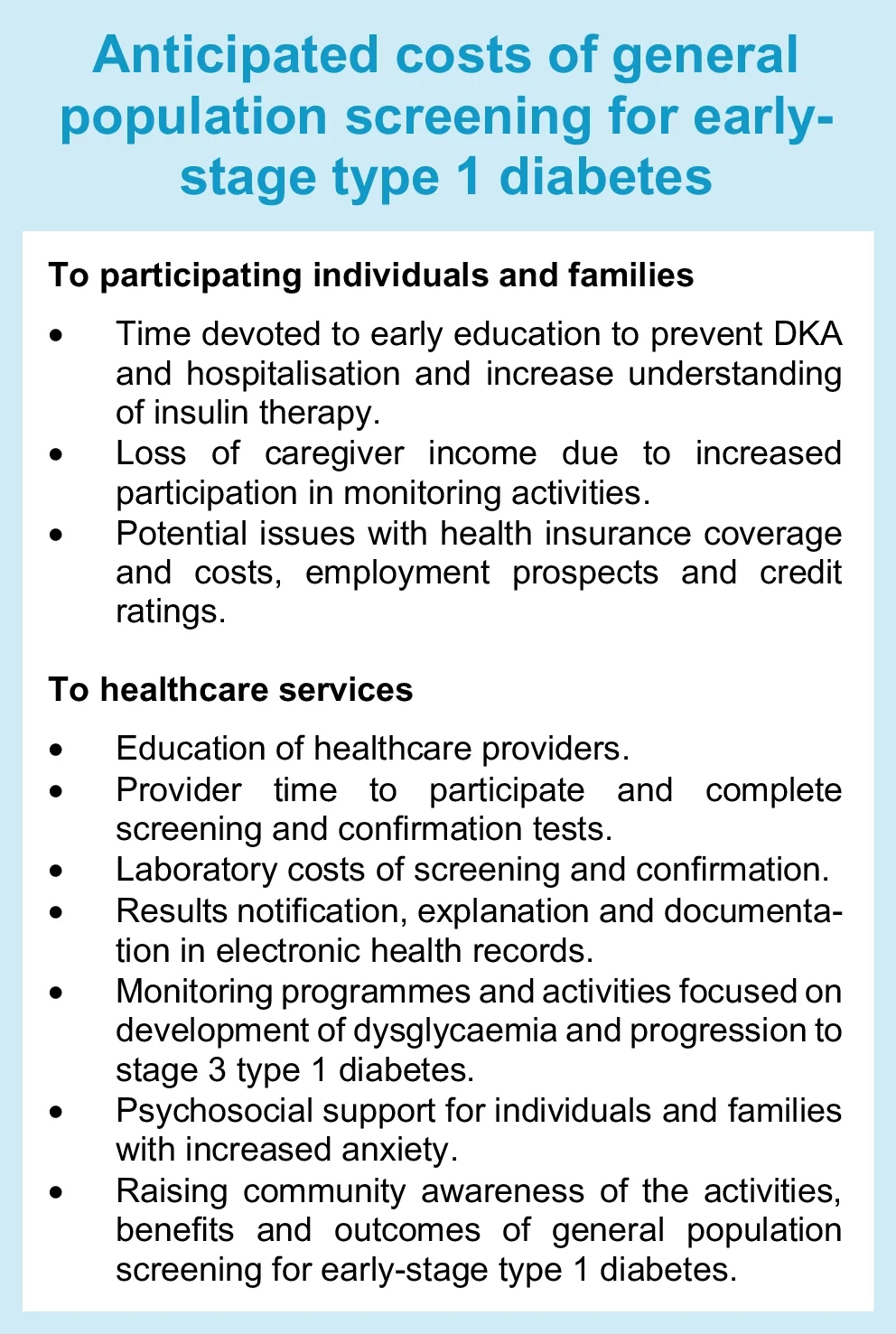

## Navigating the real world vs the ideal world

As much as possible, these consensus recommendations identify the principles and practice of general population screening for IAbs as a best-case scenario. This assumes sufficient resources are available and that healthcare stakeholders are supportive of the goals. However, we must acknowledge that the realities of initiating and implementing general population screening for early-stage type 1 diabetes are likely to differ from this ideal, given national and regional considerations. These may include the regional prevalence of type 1 diabetes and the burden of DKA, differences in healthcare systems and infrastructure, patterns of health service use, and competing health needs and priorities, as well as cultural norms. Access to resources is an important issue in this context. For example, significant variation exists regarding availability of clinical laboratory services that are licensed to undertake IAb testing at scale and the range of IAb assays available. Thus, using different assays for screening and confirmatory testing may not be feasible in some locations. Ideally, the instigation of general population screening for early-stage type 1 diabetes will prompt services to be upgraded and adapted to meet the needs of the screening programme but that may not be universal and will likely take time. Similarly, the appropriate setting for IAb screening will likely require additional training for HCPs so that appropriate information be provided to individuals and sampling is correctly undertaken. For general population IAb screening there are regional variations in consenting and data privacy issues that must be navigated, and must be resolved. A key consideration is that general population screening for IAbs will identify individuals with early-stage type 1 diabetes who need ongoing care. This will likely include participation in an organised monitoring programme, with ongoing clinical care, education and support (with further formal psychosocial intervention, where needed), access to disease-modifying therapies and/or clinical trials, as available.

## Gaps in evidence

The following gaps in evidence still exist:

- Acceptability and feasibility of adult general population IAb screening, and the recommended age for testing
- Data on the onset, frequency and progression of IAbs in adult general populations
- Predictive value of a single IAb in general population screening
- Alternative settings in addition to primary care and implementation strategies for screening and follow-up
- A standardised model of clinical care for people with early-stage type 1 diabetes with objective outcome measures
- Data on the prevalence of early-stage type 1 diabetes and disease progression in individuals of diverse racial and ethnic demographics
- Evidence for a feasible, structured patient-facing education and support programme for screening participants and individuals with early-stage type 1 diabetes.

## Strengths and limitations

A limitation of this consensus process is the relatively small number of initiatives directed towards general population screening for IAbs for detection of early-stage type 1 diabetes and in public health settings. Consequently, several recommendations are based on limited evidence or are extrapolated from research studies in children and adolescents with a higher genetic risk for early-stage type 1 diabetes. Given the scope of the recommendations, it is a limitation that there is very little available data on cost-effectiveness for general population IAb screening. In this context, a further limitation is that population-wide screening for IAbs may not be sufficiently cost-effective or warranted in countries or populations with a much lower prevalence of type 1 diabetes in children (e.g. as observed in several Asian countries). In these contexts, a more targeted screening strategy focused on at-risk populations may be more suitable. The cohorts reported in the available research studies are from North America, Europe and Australia and are under-represented for broader racial and ethnic demographics, which is a limitation, as is the fact that the author group is drawn from these geographical locations. Similarly, within the author group, only two authors (MR, LAD) are formally accredited in public health or health economics. A further limitation is that the selection of the consensus panel was not systematised, and participants’ comments in camera were not anonymous within the NGT methodology, which increases the chance of conformity bias. However, the NGT methodology involved a proactive participant contribution at the start of each virtual meeting and the consensus statements were adopted following a blinded voting step. Thus, we believe the risk of conformity bias has been minimised. A key strength of the consensus is that it reflects the clinical experience of a large international author group with expertise in the detection and management of early-stage type 1 diabetes. Another important strength is that the consensus recommendations were developed through an NGT process, a recognised methodology for building consensus through structured brainstorming, discussion and moderated debate. This process was actively managed to ensure that all opinions and contributions were heard and discussed and that the final consensus statements were agreed by all members of the working group, using a blinded voting step that ensured anonymity.

## Conclusions

Detection of type 1 diabetes can now be achieved in its early stages, when autoimmunity is present but sufficient functional beta cell mass is still available to prevent frank hyperglycaemia. Evidence from research in families already affected by type 1 diabetes and in children and adolescents at increased genetic risk and emerging studies in the general population demonstrate that detection of early-stage type 1 diabetes can be achieved effectively through IAb testing, resulting in improved outcomes including the reduction in frequency of DKA at clinical presentation with stage 3 type 1 diabetes. Whereas opportunistic screening for the presence of IAbs can be performed at any age, we propose a structured pathway of screening for IAbs in childhood, with screening initiated at age 2–4 years and rescreening of those who screened negative during childhood, at the times when other preventive services are also being offered (i.e. at age 6–8 years and 10–15 years). This consensus guidance will need to be translated into practice according to the prevailing healthcare system (and patient preference) in different national/regional settings. Adaptation of healthcare systems is recommended, to embed this screening in the established routines of primary care settings (e.g. linked to vaccination visits and well-baby examinations). It should be clear that general population IAb screening initiatives should only roll out when healthcare services are fully prepared to implement the full screening pathway, including education and metabolic monitoring of those detected with early-stage type 1 diabetes. Combined with the arrival of disease-modifying therapies, this strategy should open the way to the delay and eventual prevention of clinical stage 3 type 1 diabetes.

## Supplementary Information

Below is the link to the electronic supplementary material.

ESM Table 1 (PDF 132 KB)

## Data Availability

No new data were created or analysed in this article. All data supporting the findings of this consensus report are available within the article or from the references cited within the article.

## Abbreviations

DBS: Dried blood spot
DIPP: Type 1 Diabetes Prediction and Prevention
DKA: Diabetic ketoacidosis
ECL: Electrochemiluminescence
ELSA: Early Surveillance for Autoimmune Diabetes
HCP: Healthcare professional
IA-2: Insulinoma antigen-2
IA-2A: IA-2 autoantibody
IAb: Islet autoantibody
IASP: Islet Autoantibody Standardization Program
ICA: Islet cell antibody
NGT: Nominal group technique
NPV: Negative predictive value
PPV: Positive predictive value
RBA: Radiobinding assay
T1DI: Type 1 Diabetes Intelligence
T1DRA: Type 1 Diabetes Risk in Adults
TEDDY: The Environmental Determinants of Diabetes in the Young
