# Supplementary material for: International consensus guidance for general population screening for islet autoantibodies to diagnose early-stage type 1 diabetes: a nominal group technique process
Source: Diabetologia. 2026 Sep 10;69(12):3370–95. doi: 10.1007/s00125-026-06841-z (PMC13714164; doi:10.1007/s00125-026-06841-z)
Supplement: Supplementary file 1 [file 125_2026_6841_MOESM1_ESM.pdf]

ESM Table 1 Evidence appraisal: American Diabetes Association grading system reproduced from Table 1 ADA evidence-grading system for "Standards of Care in Diabetes"

Level of Evidence	Description
**A**	Clear evidence from well-conducted, generalizable randomized controlled trials that are adequately powered, including:<br>● Evidence from a well-conducted multicenter trial<br>● Evidence from a meta-analysis that incorporated quality ratings in the analysis<br>Supportive evidence from well-conducted randomized controlled trials that are adequately powered, including:<br>● Evidence from a well-conducted trial at one or more institutions<br>● Evidence from meta-analysis incorporated quality ratings in the analysis
**B**	Supportive evidence from well-conducted cohort studies, including:<br>● Evidence from a well-conducted prospective study or registry<br>● Evidence from a well-conducted meta-analysis of cohort studies<br>Supportive evidence from well-conducted case control study
**C**	Supportive evidence from poorly controlled or uncontrolled studies, including:<br>● Evidence from randomized clinical trials with one or more major or three or more minor methodological flaws that could invalidate the results<br>● Evidence from observational studies with high potential for bias (such as case series with comparison with historical controls)<br>● Evidence from case series or case reports<br>Conflicting evidence with the weight of evidence supporting the recommendation
**E**	Expert consensus or clinical experience

Reprinted with permission from The American Diabetes Association. Copyright 2025 by the American Diabetes Association.
